# Simple and Easy Surgical Technique for Infantile Hemangiomas: Intralesional Excision and Primary Closure

**Published:** 2015-01-15

**Authors:** Tadashi Nomura, Takeo Osaki, Hiroyoshi Ishinagi, Hirotaka Ejiri, Hiroto Terashi

**Affiliations:** ^a^Department of Plastic Surgery, Kobe University Graduate School of Medicine, Kobe; ^b^Department of Plastic Surgery, Steel Memorial Hirohata Hospital, Himeji; ^c^Department of Plastic Surgery, National Hospital Organization Himeji Medical Center, Himeji, Japan

**Keywords:** infantile hemangioma, intralesional excision, primary closure, scar, dog ear

## Abstract

**Objective:** Infantile hemangioma (IH) is a benign vascular tumor that gradually shrinks over several years. Involuting or involuted IHs usually retain their shape, however, and result in redundant skin or conspicuous scarring due to ulceration in the proliferating phase. We present a case series of 12 patients who underwent intralesional excision and primary closure for treatment of involuting or involuted IH. **Methods:** Twelve patients (5 boys, 7 girls) underwent our treatment method for involuting or involuted IH. A blinded assessor evaluated clinical result of each patient. **Results:** Surgical results were excellent in 4 patients, good in 6, and fair in 2. A small dog ear was prominent in 1 patient; nevertheless, all parents were satisfied with the results. **Conclusions:** Intralesional excision and primary closure for treatment of involuting or involuted IH is an easy and simple procedure that does not result in dog-ear formation or elongated residual scarring.

Infantile hemangioma (IH) is a benign vascular tumor that grows rapidly until the age of 1 year and then gradually shrinks over several years thereafter. Infantile hemangioma has a high incidence rate, affecting approximately 10% of children.^1^ Many interventions for IH have been reported. In particular, pulsed dye laser treatment is effective for small or superficial lesions,^2^ whereas oral steroids or propranolol treatment is recommended for problematic IHs.^3^^,^^4^ These treatments are effective for IHs in the proliferating phase. However, involuting or involuted IHs usually retain their shape and result in redundant skin or conspicuous scarring due to ulceration in the proliferating phase.^5^ In the present report, we describe a simple excision method for treatment of involuting or involuted IH based on serial excision.

## MATERIAL AND METHODS

Between 2004 and 2012, a total of 12 patients (5 boys, 7 girls) underwent surgical resection for involuting or involuted IH in the Department of Plastic Surgery of Himeji Medical Center, Himeji City, Japan. For each patient, age at surgery, lesion location, operative indication, and previous interventions were noted. Results were divided into 4 categories based on cosmetic outcome (scarring and deformity) as follows: (1) excellent, little, or invisible scarring without deformity; (2) good, visible scarring without deformity; (3) fair, visible scarring with minor deformity; and (4) poor, conspicuous scarring or deformity. Three plastic surgeons who were blinded to the treatment method independently assessed the clinical result of each patient based on patient photographs, the worst of which was adopted as the final result.

## SURGICAL PROCEDURE

**Figure 1:**
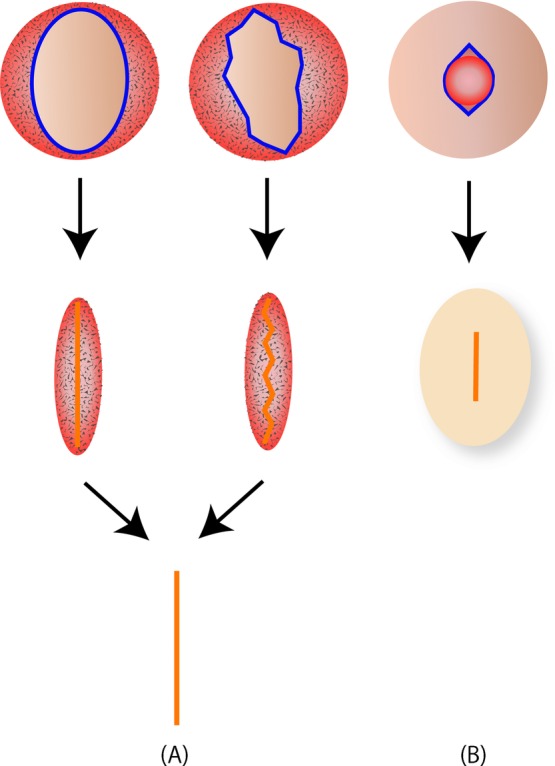
Illustrations, demonstrating intralesional excision of infantile hemangioma (IH). (*a*) Elliptical resection with dull apical angles and primary closure of IH. A zig-zag incision was selected in certain cases according to the shape of the damaged skin. (*b*) For lesions that mainly consisted of a deep component, minimal excision of the blemished skin and scooping out of the deep lesion was performed.

For lesions with thin and excessive residual skin, intralesional elliptical resection with dull apical angles and primary closure was performed as the initial procedure. If the scar following ulceration was apparent, an incisional line was drawn along the margin of the scar. Consequently, a zig-zag incision was selected in certain cases. Important aspects of the procedure were that the incisional line was made within the lesion and never across the normal skin, and that the outermost portion of the lesion was intentionally preserved. If the residual lesion was very small and limited after the first operation, we performed routine follow-up without additional treatment. If the residual lesion was large, this surgical procedure was repeated 2 or 3 times in a similar manner. At the second or third operation, the residual lesion was relatively more oval in shape. Consequently, we excised the residual lesion with a lenticular shape. The time interval between each operation was at least 3 months. For lesions that mainly consisted of a deep component, minimal excision of the blemished skin and scooping out of the deep lesion was performed. After the procedure, dog ear and operative scar formations usually were not conspicuous (Fig 1).

## RESULTS

The mean age at surgery was 38.6 months (range: 18–62 months), and the mean follow-up period from the last operation was 12.3 months. The hemangioma was located in the head and neck region in 6 cases, the trunk in 3, and the extremity in 3. The indication for surgery was an esthetic improvement in all cases. Five patients underwent flashlamp-pulsed dye laser treatment preoperatively. The mean number of surgeries was 1.5 (range: 1–3). Surgical results were excellent in 4 patients, good in 6, and fair in 2 (Table 1). A small dog ear was prominent in 1 patient (case 3); nevertheless, all parents were satisfied with the results (Table 1).

## CASE REPORTS

### Case 4

**Figure 2:**
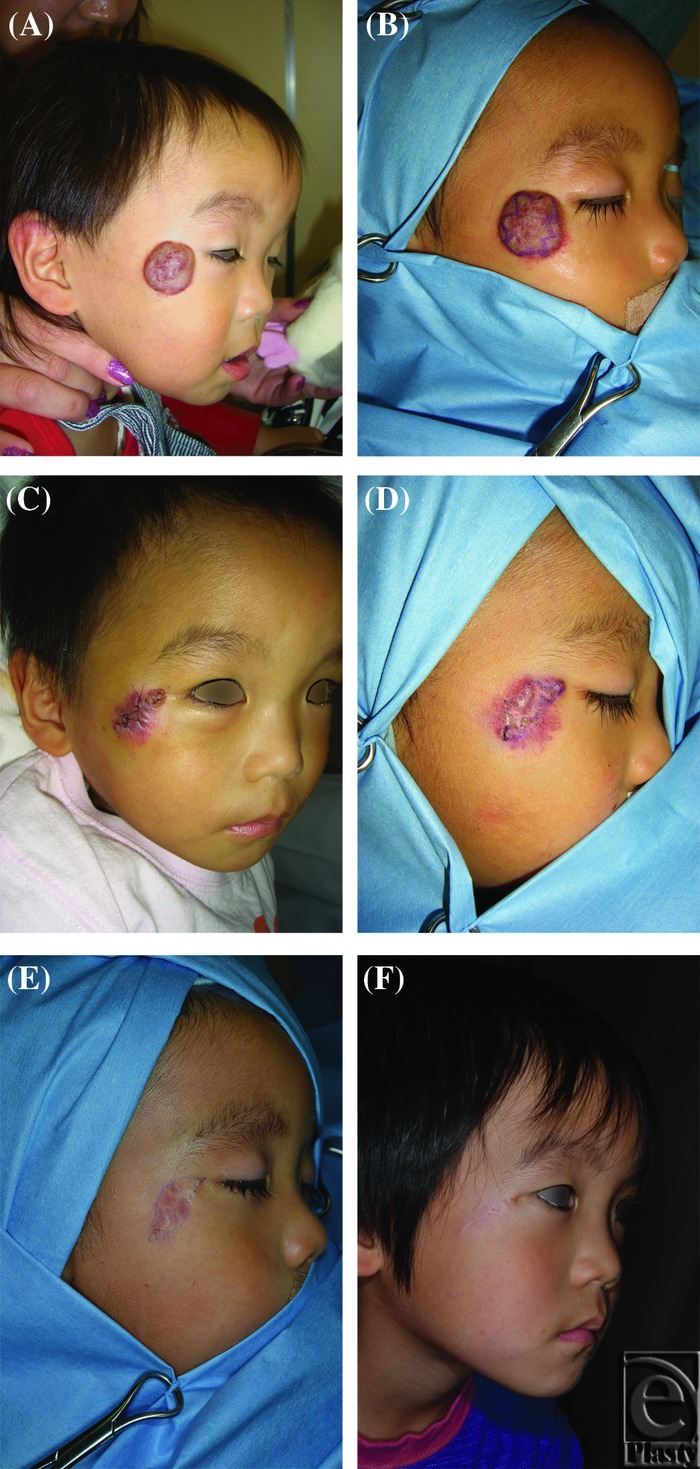
Case 4. A 23-month-old boy with a hemangioma in the right zygomatic region. (*a*) Preoperative photograph. (*b*) A zig-zag incision was selected in the first operation. Three intralesional excisions were performed. (*c*) Results at 3 days after the initial operation. (*d*) Design of the incisional line in the second operation. (*e*) Design of the incisional line in the last operation. (*f*) Results at 6 months after the last operation. The resulting scar was not conspicuous, and the outcome was judged as excellent.

A 23-month-old boy presented with a hemangioma in the right zygomatic region (Fig 2a), for which 3 intralesional excisions were performed (Figs 2b-2e). The resulting scar was not conspicuous, and the outcome was judged as excellent (Fig 2f).

### Case 11

**Figure 3:**
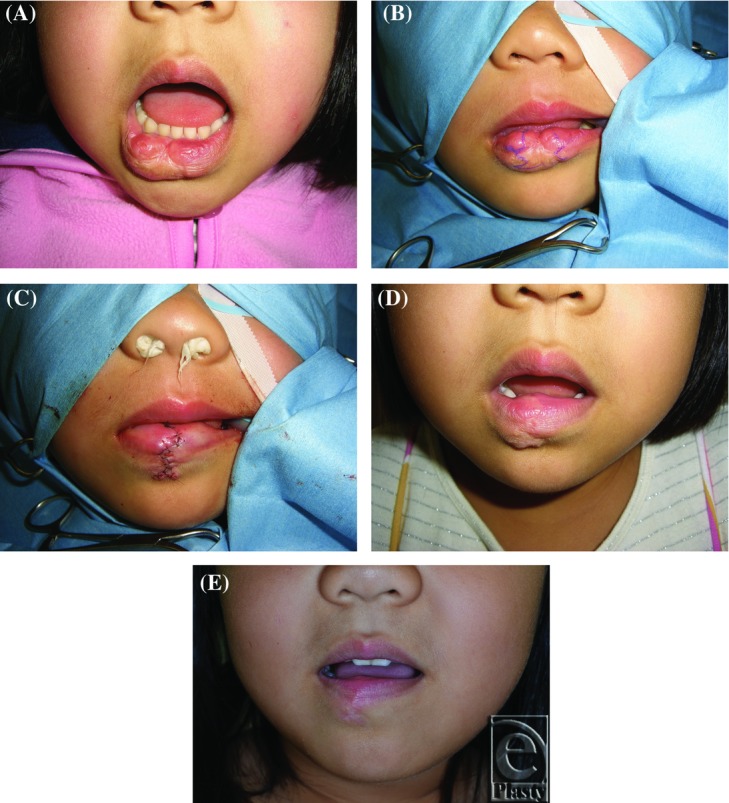
Case 11. A 55-month-old girl with a fibrofatty tissue and a scar due to a large hemangioma on the lower lip. (*a*) Preoperative photograph. (*b*) Design of the incisional line in the first operation. (*c*) Results immediately after wound closure. (*d*) Results at 6 months after the initial operation. (*e*) Results at 4 months after the second operation. The outcome was judged as good.

A 55-month-old girl presented with fibrofatty tissue and a scar due to a large hemangioma on the lower lip (Fig 3a). Two excisions were performed (Figs 3b-d), after which no distortion or functional impairment of the lower lip remained; thus, the result was judged as good (Fig 3e).

## DISCUSSION

Surgery for IH is controversial, because the lesion would involute with time. However, even with involuted lesions, the scar resulting from blistering, fibrofatty bulks, or residual excess of thin skin is occasionally esthetically problematic and can cause psychological stress to parents. Couto et al^6^ reported that most IHs do not improve significantly after 3.5 years of age and suggested that reconstructive procedures should be considered at this age. In agreement with their conclusion, we confirmed the advantages of intralesional fusiform-shaped excision as a reconstructive procedure for involuting IH in this report.

Mulliken et al^7^ reported the efficacy of circular excision and purse-strings closure for treatment of IH. Since this report was published, other authors have confirmed the usefulness of the method.^8^^,^^9^ However, this method is usually not indicated for lesions of the free margin, such as in the periorbital regions or on the upper and lower lips. A ring of pleats formation is apparent temporarily as a result of surgery. In contrast, for lenticular excision and primary closure—a conventional excisional technique for skin tumors—a 3:1 excision-to-lesion length ratio is required.^7^ Consequently, the residual scar is longer than the lesion, and terminal dog ears may be apparent. We believe that this theorem is applicable to complete excision. If partial excision is performed, dog ears will not be apparent and the scar length will not be longer than the lesion. In our series, intralesional elliptical resection with dull apical angles and primary closure was demonstrated to be a good method in that only 1 patient developed dog-ear formation and most had short scars. The reasons for these results are as follows: (1) The lesions were expanded, as noted with the tissue expansion method, into a dome-like shape, thus facilitating scooping out of the fibrofatty tissue, which caused excess, redundant skin, followed by easy closure of the wound edge without tension. (2) The expanded skin might have morphological plasticity. Therefore, the procedure is simple and easy to perform without causing pleats formation.

Klubersh et al^10^ reported good cosmetic results of staged excision and primary closure in 46 cases of IH. However, they did not discuss dog-ear formation or intralesional excision. The advantages of our method based on intralesional excision are that the residual scar is shorter than the original lesions as well as the absence of dog ears. It is important that the wound edge is closed loosely because of excess skin formation. In this study, we experienced 2 cases with fair results. The expanded skin of the lesion was not so excessive in these 2 cases. Pulling firmly toward each wound edge including nearby healthy skin might have been a factor in these cases.

In the present report, the cases described were involuting or involuted IHs, and not alarming hemangiomas, which are functionally problematic in the proliferating phase. For alarming hemangiomas, nonsurgical treatment modalities, such as corticosteroid or propranolol administration, are recommended.^3^^,^^4^ In the past, early surgical intervention for IH was suggested.^11^ In our opinion, partial excision of IHs in the proliferating phase is usually difficult, as intraoperative bleeding is more excessive and primary closure is more difficult due to the residual brittle lesion. Therefore, we avoid excising IHs in the proliferating phase. Our procedure, which is not indicated for proliferating IHs but for involuting or involuted IHs, is easy to perform and cosmetically effective.

## CONCLUSION

We present 12 cases of involuting or involuted IH treated with intralesional excision and primary closure—an easy and simple procedure that does not result in dog-ear formation or elongated residual scarring.

## Figures and Tables

**Table 1:** Clinical data of 12 patients with infantile hemangioma

Case	Sex	Age, mo	Location	Size, mm	Previous intervention	Number of surgeries	Result
1	M	41	Cheek	60 × 50	None	2	Good
2	F	48	Upper arm	25 × 40	Dye Laser	2	Good
3	F	30	Back	30 × 40	Dye Laser	2	Fair
4	M	23	Zygomatic region	20 × 20	Dye Laser	3	Excellent
5	F	49	Neck	25 × 25	none	1	Fair
6	M	27	Forearm	40 × 40	Dye Laser	1	Excellent
7	M	62	Frontal	30 × 30	none	1	Excellent
8	F	30	Chest	27 × 25	Dye Laser	1	Excellent
9	M	18	Back	25 × 60	none	1	Good
10	F	50	Parietal region	24 × 32	none	1	Good
11	F	55	Lower lip	30 × 25	none	2	Good
12	F	30	Forearm	45 × 40	none	1	Good
